# Unraveling the relative impact of material and optical stochastic effects on EUV LWR

**DOI:** 10.1038/s41598-025-29021-2

**Published:** 2025-12-09

**Authors:** Ji Young Park, Yongbeom Seo, Dong-Hun Shin, Hyunwoo Chae, Yunsu Jang, Won-Joon Son, Seungmin Lee, Heeyoung Go, ChangMin Park, Dae Sin Kim, Myung Mo Sung, Chawon Koh

**Affiliations:** 1 Semiconductor R&D Center, Samsung Electronics Co., Ltd, Hwaseong-si, Gyeonggi-do 18448 Republic of Korea; 2 Department of Chemistry, Hanyang University, Seoul, 04763 Republic of Korea; 3 Department of Material Science & Engineering, Yonsei University, Seoul, 03722 Republic of Korea

**Keywords:** Extreme ultraviolet lithography, Photon-shot noise, Stochastic effect, Photoresist, Line-width roughness, Engineering, Materials science, Optics and photonics, Physics

## Abstract

Extreme ultraviolet (EUV), with a 13.5 nm wavelength and 92 eV, produces high-resolution patterns but becomes more sensitive to stochastic effects because of its lower photon density compared to ArF and KrF, which have energies less than 10 eV. Therefore, controlling the stochastic effect is getting attention as EUV becomes mandatory to obtain ultrafine patterns. Unfortunately, various stochastic terms come from mask roughness, light-source, and photoresist materials, but each stochastic term is entangled and hard to decouple experimentally. Herein, we performed pattern shape simulation, separated each stochastic effect, and obtained patterns. Among the mask pattern roughness, photon-induced optical stochastics, and photoresist-induced material stochastics, material stochastics shows the most significant improvement on pattern break risk (2.5 nm widened failure-free window). In addition, the dispersion of photons caused by optical stochastic effects or mask pattern roughness plays a role in offsetting the excessive degradation of patterns due to material stochastic effects. Our simulation-based approach clarifies the role of each stochastic effect in pattern formation and guides process conditions and the novel photoresist development.

## Introduction

With the rapid development of artificial intelligence (AI) technology these days, chipmakers are asked to design and supply chips that can support more complex processes at high speed. This technical requirement highly demands the development of technology that can accurately implement ultra-fine patterns, resulting in rapid growth of the reliance on extreme ultraviolet (EUV) lithography^1–3^. However, due to the high- equipment cost and power consumption caused by the EUV equipment itself, it is important to improve the efficiency of the process using EUV, and the development of technology that can accurately implement ultrafine patterns is necessary as the process difficulty increases in handling sub-10 nm patterns. In short, to increase the cost efficiency of EUV-driven patterning processes, it is mandatory to extend the process margin, which guarantees high-resolution patterns without defects. In addition, high-energy photon of EUV, 92 eV, has 14 times lower photon density than KrF and ArF sources and becomes more sensitive to photon-shot noise (PSN)^4^. High-PSN sensitivity makes stochastic effects during the patterning process more critical to patterning performance. One of the most prominent manifestations of these effects is line-width roughness (LWR) and critical dimension (CD) variability, which directly affect device performance and yield^5–7^. Reducing LWR becomes a critical goal to minimize the occurrence of pattern defects such as bridges in line and space (L/S) patterns, thereby achieving the accurate target CD implementation.

1 $$LWR~\left( {nm} \right)=~{k_4} \cdot \frac{1}{{NILS}} \cdot \sqrt {\frac{{h\nu \left( {eV} \right)}}{{Dose\left( {mJ/c{m^2}} \right)}}}$$

As shown in the above Eq. (1), LWR are inversely proportional to the normalized image log-slope (NILS) and dose of EUV optics, and proportional to a constant k_4_, which incorporates all process-related image degradation effects. All process-related image degradation effects, such as material properties of photoresist and process conditions in the spinner and scanner, are included in a k_4_ and a k_4_ has been considered as constants that do not change with process conditions^8^. The NILS term is related to optical conditions that depend on pattern pitch, CD, mask, illumination system, numerical aperture (NA), out-of-band (OOB) errors, etc. NILS can be changed by process conditions and an inverse relationship with LWR. Therefore, under the same conditions, as the dose increases, LWR should be smaller as it is inversely proportional to dose. For the L/S patterns from positive-tone development (PTD) photoresist, high-dose leads to a larger space CD, the distance between the patterns, and LWR is inversely proportional to the space CD. However, P. D. Bisschop’s report^9^ and our experiments have shown that as the pitch becomes smaller, less than 42 nm, LWR is degraded again as the space CD size of the L/S pattern gradually increases. As a result, the space CD-LWR correlation graph looks like a smile-shaped, quadratic parabolic curve.

Accordingly, in the wide space CD region of small pitch, it is necessary to consider the possibility that k_4_ is not a constant but a variable due to the influence of high-dose. This dose dependency influences the stochastic distribution term at each stage until the pattern is formed^10–17^. However, in real experimental conditions, it is impossible to decouple each stochastic term because each factor is entangled. The effects—arising from the discrete nature of energy deposition, molecular interactions, and material responses—have a significant impact on pattern fidelity. Among the various sources of stochasticity, material-related stochastic effects—such as variations in photoacid generation, quencher diffusion, polymer reactivity, and development contrast—are important contributors to variability. However, despite their potential significance, many previous studies have treated material stochastic effects as a secondary factor or approximated them with constant parameters. As we mentioned before, the previous concept of a constant k_4_ value may be insufficient, particularly in the ultra-fine pattern pitches, where the local distribution and interaction of reactants can dominate pattern outcomes.

To this end, we present a simulation-based framework to decouple and analyze the role of optical and material stochastic effects in pattern formation. By incorporating spatially and temporally varying material parameters into a stochastic lithography model, we were able to reproduce experimental trends of defects and LWR, which show a smile-shaped curve that emerges specifically at 36 nm pitch. Our results suggest that controlling material stochasticity—through resist formulation, quencher design, or post-exposure bake tuning—may be key to improving pattern fidelity and suppressing defectivity in sub-10 nm resolution lithography. Furthermore, since direct experimental isolation of material stochasticity is challenging due to convolution with other noise sources, our simulation approach offers a novel pathway to quantify and visualize these effects. This work provides new insight into stochastic pattern degradation mechanisms and suggests concrete directions for materials and process optimization in next-generation lithographic technologies. Unless otherwise specified, the analysis throughout this paper focuses on positive-tone photoresist. CD refers to space CD, which denotes the width of vacant spaces between patterns. LWR represents an unbiased LWR (ULWR), which removes white noise from the measurement method of the original LWR values.

### Optical and material stochastic effects

**Fig. 1:**
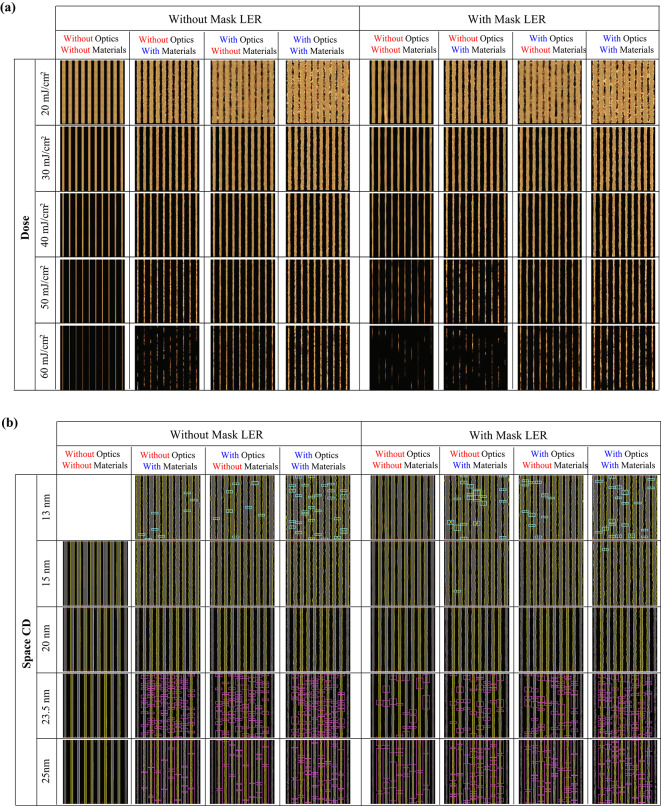
(**a**) 36 nm pitch L/S pattern with optical stochastic effects, material stochastic effects, and mask LER effects. (**b**) Defect detection for CDs of 12, 15, 23.5, and 25 nm. Cyan and magenta colors indicate bridge and break defects, respectively.

We conducted pattern shape modeling with and without considering mask pattern roughness, photon and secondary electron-induced optical stochastic effects, and material stochastic effects. We obtained pattern shapes and defect occurrence frequencies, which are presented in Figure 1, for each condition where the stochastic effect was turned on (with stochastics) and turned off (without stochastics). Under various stochastic conditions, patterns within a dose range of 20 mJ/cm² to 60 mJ/cm² are shown in Figure 1a, and patterns within a CD range of 13 nm to 25 nm are shown in Figure 1b. In Figure 1b, bridge defects are marked as a cyan-colored box, and break defects are marked as a magenta-colored box.

**Table 1.**
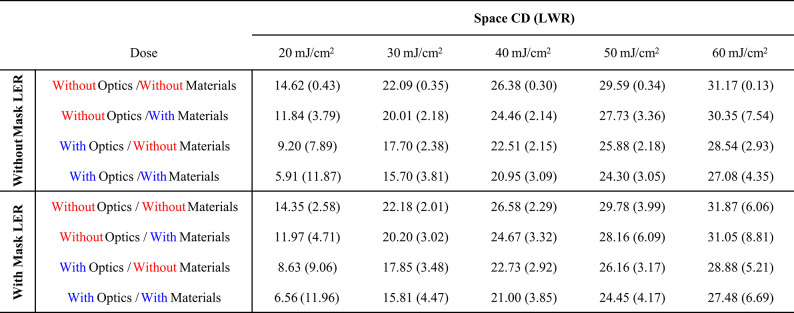
Space CD (nm) and LWR (nm) on each stochastic effect condition and the dose variation through 20 mJ/cm^2^ to 60 mJ/cm^2^

Detailed pattern quality is summarized in Table 1. To focus on optical and material stochastic effects, we only consider the case without mask LER in this section. When there is no stochastic effect, LWR is rarely changed and only have values around 0.3 nm. The randomness of polymer chain length causes small amount of non-zero LWR, as we only controlled acid diffusion in material stochastic effects. When only material or optical stochastic effects is included, the LWR at dose 40 mJ/cm² is nearly the same (2.14 nm and 2.15 nm for each). However, when the dose decreased from 40 mJ/cm^2^ to 20 mJ/cm^2^, material stochastic effects cause LWR degradation from 2.14 nm to 3.79 nm, while optical stochastic effects cause larger degradation from 2.15 nm to 7.89 nm when mask LER is not considered. Otherwise, when the dose increases from 40 mJ/cm^2^ to 60 mJ/cm^2^, opposite degradation behaviors are observed. material stochastic effects cause LWR degradation from 2.14 nm to 7.54 nm, while optical stochastic effects cause less LWR degradation from 2.15 nm to 2.93 nm. At the high dose region, around 60 mJ/cm^2^, an interesting feature is observed. When both stochastic effects are included together, the LWR degradation is smoother than the case when only the material stochastic effects are included. This point will be discussed in the following section.

**Fig. 2:**
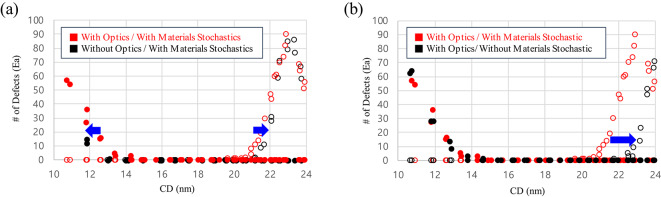
The CD–defect relationship for a 36 nm pitch L/S pattern with optical and material stochastic effects. Red dots indicate the case with both effects, and black dots indicate the cases with only (**a**) optical stochastic effects (material stochastic effects are removed) or (**b**) material stochastic effects (optical stochastic effects are removed). The blue arrows show the defect margin change when each effect is removed.

In Fig. 2, the CD versus defect trend was analyzed based on the data in Fig. 1b to verify the patterns. Each dot represents the number of bridge (filled) or break (unfilled) defects counted in a single simulation image. Where the bridge/break defect number equals 0, it can be defined as a failure-free window. We define bridge margin as the CD point where the number of defects are counted over than 0, because it means that the defect probability is not 0 from that point. The break margin can be defined in the same way as the non-zero start point of the unfilled dots. As shown in Fig. 2a, when the optical stochastic effects are off and the material stochastic effects are on, the bridge margin shifts left from 14.3 nm to 13.1 nm, and the break margin shifts right from 19.9 nm to 21.2 nm compared with when all the stochastic effects are turned on. As the bridge margin shifts to the left and the break margin shifts to the right, the failure-free window becomes wider, and we can expect the more flexible process condition to obtain the target CD. On the other hand, in Fig. 2b, the break margin greatly improves by 2.5 nm (from 19.9 nm to 22.4 nm) when the material stochastic effects are turned off, despite a slight degradation of the bridge margin from 14.3 nm to 14.6 nm. This small amount of the bridge margin degradation is caused by the fact that acid diffusion no longer covers the unexposed region at the low-dose/small CD region when material stochastic effects are turned off.

**Fig. 3:**
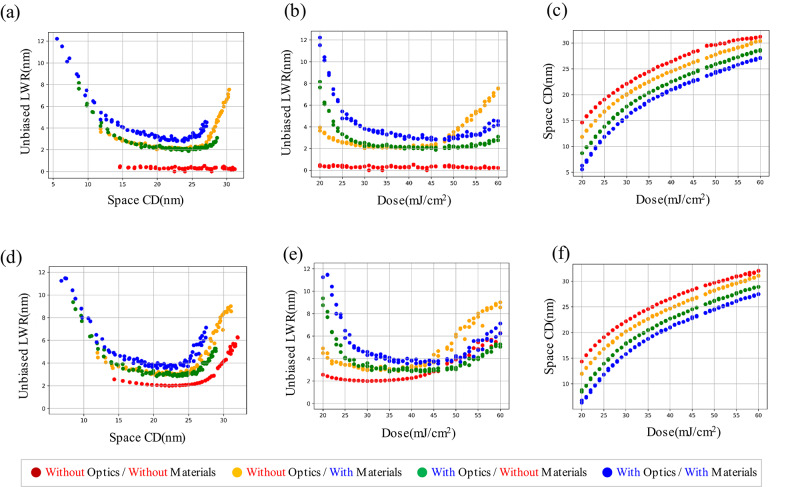
The correlation between (**a**) CD-LWR, (**b**) dose-LWR, (**c**) dose-CD without mask LER effects, and (**d**) CD-LWR, (**e**) dose-LWR, (**f**) dose-CD with mask LER effects. Without optical/with material stochastic effects (red), with optical/without material stochastic effects (yellow), without optical/with material stochastic effects (green), with optical/with material stochastic effects (blue).

Results from Fig. 2a and 2b which indicate that optical stochastic effects mainly affect the bridge margin, while material stochastic effects mainly affect the break margin can be a clue for origin of smile-shaped curve of the CD-LWR correlation. To verify the origin of the smile-shaped curve, we plotted space CD-LWR, dose-LWR, and dose-space CD correlation graph in Fig. 3 based on the patterns obtained from Fig. 1. Within the single SEM-like image obtained from the simulation, we obtained the average LWR value calculated from eight lines in the measured L/S patterns and represented it as a dot. Among four different conditions, the red points in Fig. 3 represent the case when all stochastic effects are turned off and LWR has a value around 0.3 nm across all doses and CD regions as shown in **Table 1**. Otherwise, within the CD region 13–25 nm in Fig. 3a, LWR caused by material stochastic effects (yellow dots, the optical stochastic effects are turned off) and optical stochastic effects (green dots, the material stochastic effects are turned off) show the similar values. But, in the overall region, the optical stochastic effects have an inverse proportional correlation with CD and dose values, as discussed earlier through the formula. On the other hand, the LWR curve sharply degrades at the large CD value at the high-dose region for the case where only the material stochastic effects are included. As a result, a smile-shaped curve trend where both ends degrade sharply relative to the target CD, resembling reality when both stochastic effects are turned on. Based on the results, it seems that optical stochastic effects-caused degradation is dominant at lower doses and in small CD regions, whereas material stochastic effects-caused degradation is dominant at higher doses and in large CD regions.

### Mask LER effects

**Fig. 4:**
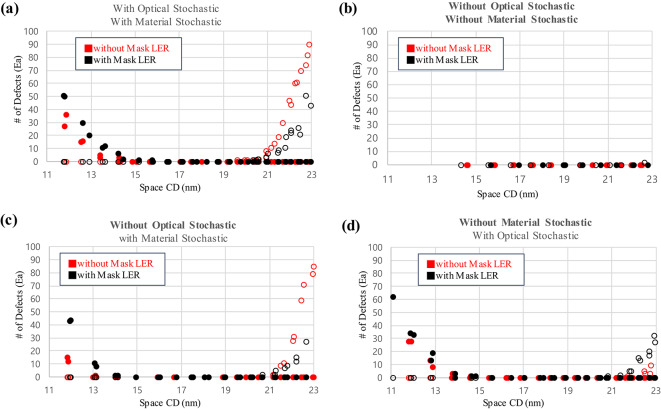
CD versus defects relationship with and without mask LER. (**a**) when all stochastic effects are included, (**b**) when all stochastic effects are eliminated, (**c**) when optical stochastic effects are eliminated, (**d**) when material stochastic effects are eliminated.

By comparing Fig. 3a, 3b, and 3c which excludes the mask LER effect, with Fig. 3d, 3e, and 3f which includes the mask LER effect, it is clear that the red dots are shifted to up and are degraded exponentially as the CD increases when the only mask LER is included (Fig. 3d) compare with nothing is included (Fig. 3a). The changed CD-LWR correlation is interpreted as caused by mask LER inducing scattered photon adsorption at the pattern edges. In addition, we examined the defect frequency-based failure-free window analysis to investigate the bridge and break margin shift according to the with or without mask LER and the removal of stochastic effects through Fig. 4; Table 2. First, considering both optical and material stochastic effects (Fig. 4a), the bridge margin degradation of 0.8 nm (right shift) is observed, and the break margin improvement is about 0.6 nm (right shift) when the mask LER effect is included. It results in the process margin narrowing only about 0.2 nm, from 5.6 nm to 5.4 nm. This is aligned with Jonckheere’s work^18^ on pattern failure analysis of various defect origins from mask. They also concluded that the failure rate is rarely changed, though they used more extreme conditions on mask absorber’s LER, σ = 1.5 nm, compared with our simulation. Figure 4b shows no defect within the range of 14 to 23 nm.

**Table 2.**
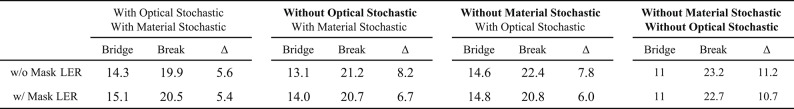
Occurrence start point of bridge/break defects and defect frequency-based failure free CD region width (Δ) (unit: nm)

On the other hand, when the optical stochastic effects are turned off and only the material stochastic effects are turned on (Figure 4c), as the mask LER effect is included, the bridge margin is degraded by 1.1 nm right shift, while the break margin is degraded by 0.5 nm left shift. As a result, the process margin becomes narrower by 1.5 nm (8.2 nm to 6.7 nm). The absence of an optical stochastic effects result in an uniform distribution of photons in the photoresist, which depends solely on scattering from the mask pattern and the random behavior of acid diffusion. Next, when the material stochastic effects are turned off and only the optical stochastic effects are turned on (Figure 4d), the 0.2 nm right shift of bridge margin and 1.6 nm left shift of break margin are observed when the mask LER effect is considered. As a result, the process margin becomes narrower, from 7.8 nm to 6.0 nm. However, we want to point out that the analysis was performed under extreme conditions using a value about 3 times larger than the typical mask LER values, around 1 nm. Therefore, the degree of degradation caused by the mask is expected to be less than the improvement due to material stochastic effects.

**Fig. 5:**
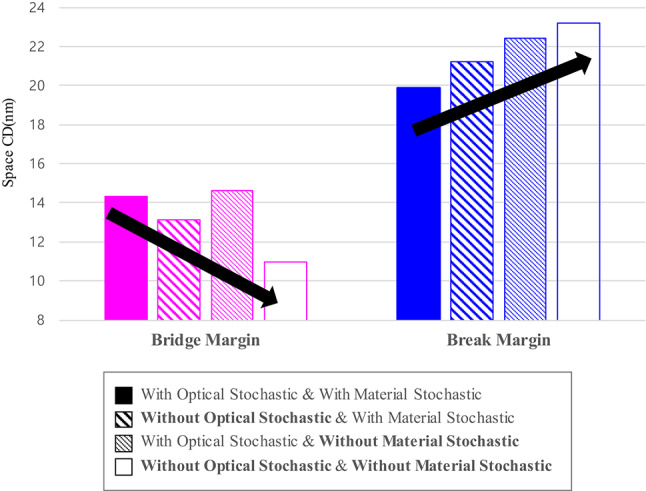
Starting point of bridge and break defect occurrence with and without optical and material stochastic effects, without considering mask LER (nm).

Through Fig. 5, we can see that the starting point of bridge defects occurrence decreases, and the starting point of break defects occurrence increases as each stochastic effect is included sequentially under without consideration of the mask LER. From left to right, stochastic effects are removed, and the bridge margin becomes smaller while the break margin becomes higher. As the break margin widening is the largest when the material stochastic effect is turned off, developing photoresists that can suppress acid diffusion and securing optimal process conditions can widen the break margin, reducing the risk of defects and improving process difficulty.

### LWR improvement at high-dose region

In Fig. 1; Table 1, it is easily acceptable that LWR degrades more when both optical and material stochastic effects are included compared with the case where only one of the stochastic effects is included. At a dose of 40 mJ/cm^2^, the LWR value increases from 2.14 nm to 3.09 nm when optical stochastic effects are added to material stochastic effects. Otherwise, when a higher dose, 60 mJ/cm^2^, is exposed, LWR decreases from 7.54 nm to 4.35 nm as the optical stochastic effects are added to the material stochastic effects. Similar to the LWR improvement, the space CD becomes smaller, and the pattern appears clearer as the bar CD becomes thicker.

**Fig. 6:**
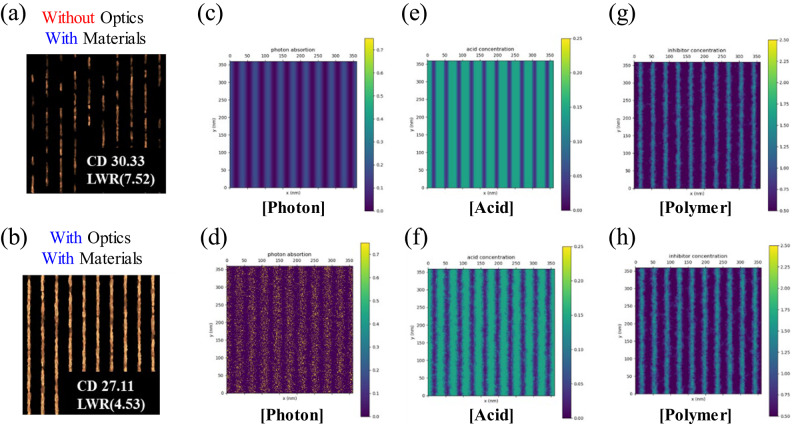
Pattern and particle distribution images at 60 mJ/cm^2^ only with materials stochastic effects (**a**), (c), (e), (g) and with both stochastic effects (**b**), (d), (f), (h). From left to right, the distribution of activated photons (**c**), (**d**), activated PAG (**e**), (**f**), and the density of remaining polymer (**g**), (**h**).

To interpret this, we visualized and analyzed the density distributions of exposed photons, activated acid, and remaining polymer in each photolithography process, specifically exposure and post-exposure bake (PEB). For a 60 mJ/cm^2^ dose, the CD is 30.33 nm and LWR is 7.52 nm when only the material stochastic effects are turned on and the optical stochastic effects are turned off (Fig. 6a). However, the CD is reduced to 27.11 nm and LWR to 4.53 nm at the same dose when both the optical and material stochastic effects are turned on (Fig. 6b). Comparing Fig. 6c and 6d, the photons have a uniform and low density (0.2) when the optical stochastic effects are turned off (Fig. 6c). At the same time, high-density (0.7) photons are scattered when both the stochastic effects are turned on (Fig. 6d). Therefore, there are some regions where photons are not absorbed. Subsequently, at the point where photons are absorbed, secondary electrons are generated, and from there, the photo acid generator (PAG) is activated and generates acid. In Fig. 6e, a wide and uniform distribution of acid is observed due to the uniform photon distribution when the optical stochastic effects are turned off. Otherwise, in Fig. 6f, some of the PAGs in the exposed area fail to capture photons and are not activated, and less acids are generated when both stochastic effects are turned on. Next, the protecting groups of the polymer are dissociated by acid, creating a difference in solubility in the developer. Compared with Fig. 6e and 6g, less polymers are deprotected in Fig. 6f and it increases the thickness of the remaining polymers (Fig. 6h) and makes a clearer pattern.

In short, at high dose, the optical stochastic effect scatters photons, reduces the PAG activation amount, and ultimately leaves a more polymer pattern which results a thicker bar CD a narrower space CD, and relatively less deteriorated LWR. Therefore, the optical stochastic effect inversely suppresses the degradation caused by material stochastic to some extent.

## Conclusion

We analyzed the contribution of optical stochastic effects, material stochastic effects, and mask LER effects on LWR of patterns in EUV patterning through simulation, as it is challenging to decouple these effects experimentally. Our study on the LWR degradation behavior of small pitch (36 nm pitch) is meaningful in guiding the lower limit caused by each stochastic effect and suggesting a direction to avoid defects in the patterns. We revealed that the smile-shaped CD-LWR correlation at 36 nm pitch L/S pattern is caused by optical stochastic effects at the low-dose/small CD region while material stochastic effects are dominant at the high-dose/large CD region. On the other hand, the LWR degradation is not simply calculated as the sum of the degradation from optical and material stochastic effects because the effects compensate each other. For example, in the case of high dose exposure, photon adsorptions can be highly localized by optical stochastic effects and which mitigates excessive acid formation and leads to improve LWR for the case where all the stochastic effects are included, compared with the case where only the material stochastic effects are considered. On the other hand, mask LER scatters the density of photon absorption and degrades patterns. We also show that 0.3 nm LWR can be achieved theoretically when both optical and material stochastic effects are turned off. However, in real process conditions, optical and material stochastic effects cannot be completely excluded in the system, and they cause a lower limit as much as the for LWR around 3 nm at the target CD and dose following our simulation result. Lastly, the defect frequency-based failure-free window analysis indicates that controlling the material stochastic effect can significantly improve the break margin at the high-dose region. In other words, controlling the material stochastic effects are the most important to reducing pattern breakage, which causes metal line bridge issues in semiconductor devices. We believe our approach would be helpful for understanding comprehensive stochastic effects in EUV patterning at small pitch and therefore improving lithography process quality.

## Methods

To generate the photoresist pattern through simulations, we obtained a mask graphic design system (GDS) for 36 nm pitch (18 nm half-pitch) L/S patternusing Synopsys’ S-LITHO software^19^. Using the GDS, we obtained aerial images of 10-line 36 nm pitch L/S pattern  (360 nm × 360 nm) with a photoresist thickness of 350 Å. In order to add mask LER effect on the mask absorber, we modified mask GDS and performed same aerial modeling under around three times harsh conditions, σ = 1, α = 0.5, and correlation length = 5 nm, compare with common mask LER. Obtained aerial images are applied to the in-house photoresist pattern simulator adapted from reference^20^.

To describe the spatial profile of secondary electrons generated from an absorbed photon, we use Eq. (2), the Bethe equation^21^. After that, we apply the Eq. (3), the Fokker–Planck equation^22^, to model the drift and diffusion behavior of secondary electrons.

2 $$\lambda =\frac{E}{{E_{p}^{2}\left[ {\beta \ln \left( {rE} \right) - \frac{C}{E}+\frac{D}{{{E^2}}}} \right]}}$$

3 $$\frac{{\partial P}}{{\partial t}}= - \nabla \cdot \left[ {\vec {A}\left( r \right)P\left( {r,t} \right)} \right]+{\nabla ^2}\left[ {D\left( r \right)P\left( {r,t} \right)} \right]$$

In Eq. (2), $\:\lambda\:$ represents the flight length of secondary electrons, E represents electron energy, and $\:\beta\:,\:C,\:and\:D$ are polymer-dependent parameters. Also, the first term in Eq. (3) represents the drift of electrons, while second term corresponds to the diffusion term, which is related to stochastic blur. Here, $\:D\left(r\right)$ denotes diffusion coefficient, which depends on temperature. Based on these equations, the optical stochastic effect is eliminated by not using the Bethe equation and only considering acid generation from the photon absorption center.

Next, we used a pre-optimized photoresist parameters of an organic chemically amplified resist (CAR), which is used in foundry processes at 36 nm pitch L/S. Based on the Kozawa model^21,23,24^, our in-house module solves the Smoluchowski equation^25^ and the point spread function (PSF)^26^ to describe the sequential process of secondary electron generation, initial protons generation from the photo acid generators (PAGs), and acid diffusion and distribution during the exposure process. After the acid distribution is obtained from previous step, it diffuses and reacts with remaining photo-decomposable quencher (PDQ) and inhibitor during the post-exposure bake (PEB) step. The PEB process described by the reaction and diffusion model is simulated with the finite difference method. Herein, we control the stochastic effects in our module via modifying the Smoluchowski equation, Eq. (4), adjusting the stochastic parameter, $\:\rho\:$, which allows control over particle probabilistic distribution and diffusion.

4 $$\frac{{\partial \rho }}{{\partial t}}=\nabla \cdot \left( {D\nabla \rho +~\chi \rho \nabla \phi } \right)$$

5 $$\rho \left( {acid} \right)=\left( {1 - r\left( {acid} \right)} \right) \cdot {\rho _c}\left( {acid} \right)+~r\left( {base} \right) \cdot {\rho _p}\left( {acid} \right)$$

6 $$\rho \left( {base} \right)=\left( {1 - r\left( {acid} \right)} \right) \cdot {\rho _c}\left( {base} \right)+~r\left( {base} \right) \cdot {\rho _p}\left( {base} \right)$$

Within the Eq. (5) and Eq. (6), $\:{\rho\:}_{c}\left(acid\right)$ and $\:{\rho\:}_{c}\left(base\right)$ represent the continuum distribution, and $\:{\rho\:}_{p}\left(acid\right)$ and $\:{\rho\:}_{p}\left(base\right)$ represent the stochastic distribution under Monte Carlo simulation. r(acid) and r(base) are the mixing ratio, values between 0 and 1, and we set r(acid) and r(base) as 0 while to remove the stochastic distribution while set r(acid) and r(base) as 1 to fully consider stochastic effects. The polymer protecting group’s deprotection is calculated by pre-set deprotection rate at the acid diffusion region. Finally, the de-protected polymer density is obtained based on the pre-set dissolution rate with the MACK4 dissolution rate model^27^.

For subsequent analysis, an in-house Python code was developed to extract SEM-like images, which were then analyzed using MetroLER 4.1 software^28^, similar to an actual SEM analysis. LWR and defect analys were conducted on these SEM-like images obtained through this process. We calculated LWR under bridge/break specifications for L/S 36 nm pitch as bridge risk starts from narrower than 11 nm space width, break risk starts from wider than 10 nm space width, which are the referencefor an actual SEM image analysis to count defects. Additionally, the changes in process margins for each stochastic effects were analyzed using the defect frequency-based modified failure-free window analysis, the CD Cliff methodology [29].

## Data Availability

The authors declare that the data supporting the findings of this study are available within the paper. Should any raw data files in another format be needed, they are available from the corresponding author (Ji Young Park) upon reasonable request.
